# Long-term follow up of Hodgkin lymphoma

**DOI:** 10.18632/oncotarget.24392

**Published:** 2018-02-03

**Authors:** David Perez-Callejo, Lorea Zurutuza, Ana Royuela, Maria Torrente, Beatriz Núñez, Virginia Calvo, Miriam Méndez, Fernando Franco, María Auxiliadora Brenes, Juan Cristobal Sánchez, Mariano Provencio

**Affiliations:** ^1^ Medical Oncology Department, Hospital Universitario Puerta de Hierro-Majadahonda, Madrid, Spain; ^2^ Statistics Department, Hospital Universitario Puerta de Hierro–Majadahonda, Madrid, Spain

**Keywords:** Hodgkin lymphoma, survival, standardized mortality ratio

## Abstract

**Background:**

Hodgkin lymphoma (HL) is the paradigm of curable disease. This study analyzed the overall survival (OS) of patients with HL and compared their survival between decades and with the expected survival of a general population.

**Results:**

The median follow-up was 22 years. The median OS was 33 years. The incidence mortality rate for all causes is 2 per every 100 patients per year. The OS of our cohort at 10 years from diagnosis was 76% (95% CI: 72–79) and 52% at 30 years (95% CI: 48–57). Overall SMR (1980–2013) was 2,943 (95% CI: 2,518–3,439). Excluding the primary tumor as the cause of death, the SMR obtained is 2,266 (95% CI: 1,895–2,710). The SMR for those patients diagnosed before the year 2000 was 2,097 (95% CI: 1,732–2,539); and for those diagnosed after 2000 was 5,218 (95% CI: 8,655). The group of patients diagnosed after 2000 had statistically significant more advanced stages, were older and less responsive to treatment.

**Conclusions:**

Despite the advances achieved, the risk of death remains higher than in the general population, mainly for those patients diagnosed after year 2000, even after almost 40 years of follow-up. This data might suggest a shift to more aggressive forms of disease in recent years.

**Patients and methods:**

A total of 595 patients diagnosed with HL were included between January 1966 and February 2014. The standardized mortality ratio (SMR) was analyzed using the annual rate of mortality in the general Spanish population, adjusted for age, sex and time period.

## INTRODUCTION

Hodgkin lymphoma (HL) is the paradigm of curable disease within the field of Medical Oncology, along with germ cell tumors. Globally, 65950 new cases of HL are diagnosed each year, accounting for 0.5% of all cancer diagnoses [1]. It is therefore considered a rare disease. In Spain, this disease has an incidence rate higher than that of the world population (2.1 and 2.5 cases per 100,000 persons-year in women and men, respectively, in Spain, compared to 0.7 and 1.1 cases worldwide) [2]. Besides, the current Spanish trend is that new diagnoses are increasing (12.5–14.7% more), according to the Spanish Network of Cancer Registries (REDECAN) [3].

In recent decades, due to the improvement in diagnostic techniques, advances in molecular biology and in the development of therapeutic options, the survival of these patients has been significantly increased [4]. This fact leads to the need to monitor the occurrence of late complications, such as the appearance of second tumors or cardiovascular events, with a consequent impact on the morbidity and mortality of these patients [5]. However, there is little information regarding the impact of new treatments regarding late toxicities, adapted to the patient’s risk, with less extension in the radiotherapy field and shorter chemotherapy schemes.

The main objective of this study was to analyze the overall survival (OS) of patients with HL and to compare it with the expected survival in the general Spanish population, with a long follow-up and search for a possible improvement in survival in the last decades of treatment, and whether there has been any change in long-term morbidity and mortality.

## RESULTS

### Patient characteristics and survival analysis

Out of a total of 624 patients diagnosed with HL, 29 patients diagnosed prior to 1975 were excluded due to a lack of epidemiological population data prior to that date. Patient characteristics are described in Table 1.

**Table 1:** Patient demographics

Characteristics	Valid *N*	*N* (%)
Sex	594	
Male		366 (61.62)
Female		228 (38.38)
Age at diagnosis (years)	590	
≤30 years		308 (52.20)
31–59 years		238 (40.34)
≥60 years		44 (7.46)
Year of diagnosis	591	
<2000		495 (83.76)
≥2000		96 (16.24)
Ann Arbor stage	585	
I		103 (17.61)
II		269 (45.98)
III		137 (23.41)
IV		76 (12.99)
Hodgkin lymphoma subtypes	589	
Nodular sclerosis (NS)		271 (46.01)
Mixed cellularity (MC)		196 (33.28)
Lymphocyte-rich (LR)		41 (6.96)
Lymphocyte depleted (LD)		51 (8.66)
Non-Specified (NS)		30 (5.09)
Response to treatment	573	
Complete		532 (92.84)
Non complete		41 (7.16)
Relapse	558	
Yes		174 (31.18)
No		384 (68.82)
Secondary tumor development	516	
Yes		106 (20.54)
No		410 (79.46)
Exitus	591	
Yes		259 (43.82)
No		332 (56.18)
Cause of death	246	
Primary tumor		56 (22.76)
Secondary tumor		56 (22.76)
Other causes		134 (54.47)

The median follow-up was 22 years (p25-p75: 8–30 years) for the entire cohort and 29 years (p25-p75: 17–33 years) for disease-free alive patients.

**Figure 1:**
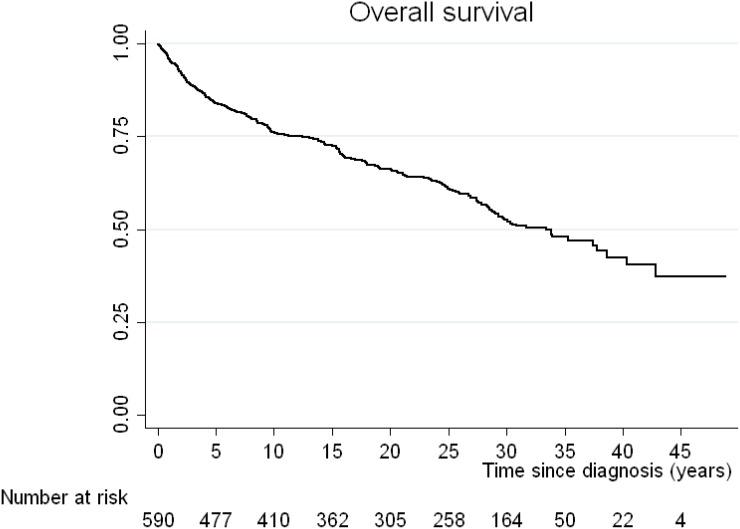
Kaplan-Meier curve for overall survival (OS) of the cohort

**Figure 2:**
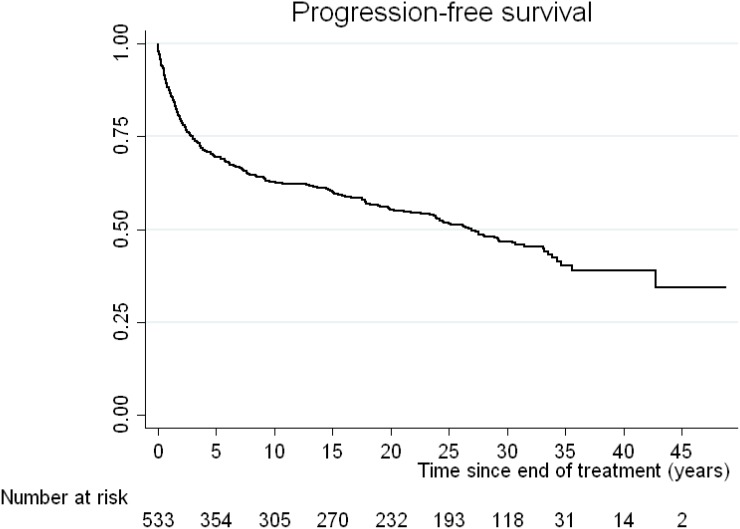
Kaplan-Meier curve for progression-free survival (PFS) of our cohort

**Figure 3:**
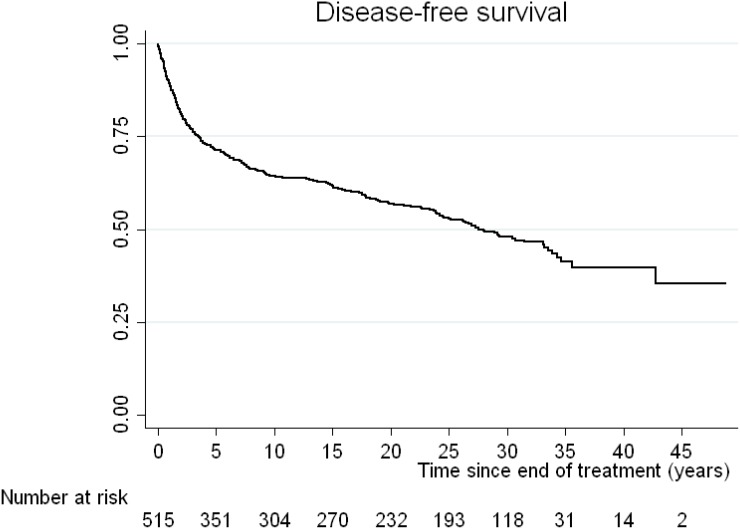
Kaplan-Meier curve for disease-free survival (DFS) of those patients with a complete response after first line treatments

The median OS was 33 years. The incidence mortality rate for all causes is 2 per every 100 patients per year. The OS of our cohort at 10 years was 76% (95% CI: 72–79), at 20 years 66% (95% CI: 62–70), at 30 years 52% (95% CI: 48–57) and at 40 years 42% (95% CI: 36–49%) (Figure 1). The PFS at 10 years was 63% (95% CI: 58–67), at 20 years 55% (95% CI: 51–60), at 30 years 46% (95% CI: 41–51), and at 40 years 39% (95% CI: 33–45%) (Figure 2). The DFS for those patients with a complete response at 10 years was 64% (95% CI: 60–68), at 20 years 57% (95% CI: 52–61), at 30 years 48%: 43–52) and at 40 years 40% (95% CI: 34–47%) (Figure 3)

### Comparison between mortality rates by study group and general population

SMR was used to quantify the increase or decrease in mortality of the study cohort compared to the general Spanish population. Overall SMR, including all-cause death, was 2,943 (95% CI: 2,518–3,439), indicating that patients with HL triple on average the risk of death compared to the general population.

**Figure 4:**
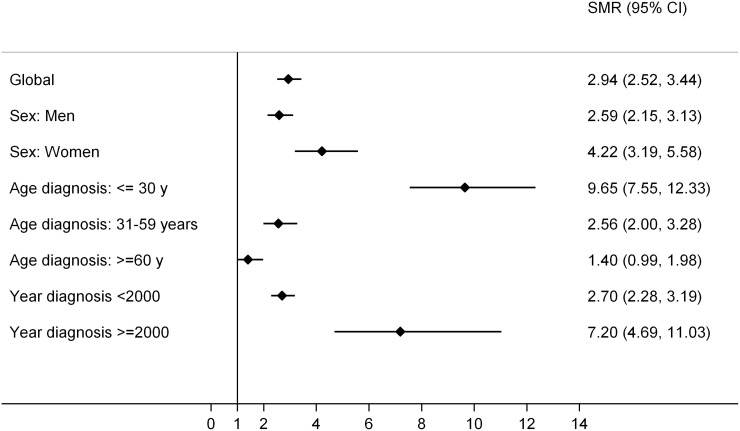
Forest plot of standardized mortality ratios (SMR), global and by subgroups The forest plot was generated through “Distiller SR Forest Plot Generator from Evidence Partners”.

Stratifying by sex, women had a higher mortality rate than men, with an SMR of 4,220 (95% CI: 3,119– 5,584), compared to 2,590 (95% CI: 2,147–3,125), respectively (Figure 4). Regarding the analysis of the different age groups at diagnosis, those patients belonging to the younger age group (≤30 years) presented a significantly higher SMR than the other groups, with an SMR of 9,653 (95% CI: 7,555–12,333). In patients between 31 and 59 years of age, SMR was 2,560 (95% CI: 1,996–3,283) and in the population over 60 years of age it was 1,400 (95% CI: 0.991–1.980), finding no differences in mortality compared to the general population.

Regarding the analysis according to the year to the diagnosis, for those patients with HL diagnosed before the year 2000, the SMR was 2,698 (95% CI: 2,282–3,190); while for patients diagnosed after the year 2000 it was 7,195 (95% CI: 4,691–11,035).

Excluding the primary tumor as the cause of death, the SMR obtained is 2,266 (95% CI: 1,895–2,710). If the cohort is divided according to the year of diagnosis of HL, the SMR for those diagnosed before the year 2000 was 2,097 (95% CI: 1,732–2,539); and for those diagnosed after 2000 was 5,218 (95% CI: 3,146–8,655).

### Adjusted analysis

A multivariable Cox regression was performed to jointly assess the association of gender, age at diagnosis, and year of diagnosis (before and after the year 2000) with all-cause mortality in our cohort. Results are shown in Table 2.

**Table 2:** Multivariable Cox regression for all-cause mortality

Variables	HR (CI 95%)	*p*
Sex (male)	1.335 (1.028– 1.732)	0.030
Age at diagnosis		
≤30 years	Ref. cat.	---
31–59 years	1.224 (0.938–1.596)	0.136
≥60 years	4.380 (2.983–6.430)	<0.001
Year of diagnosis (≥ 2000)	1.225 (0.794–1.889)	0.359

HR: Hazard Ratio; CI: Confidence Interval.

The variables associated with all-cause mortality were sex (in males, HR = 1.33, 95% CI 1.03–1.73, *p* = 0.030) and being over 60 years old (HR with respect to under 30 years = 4.38; % 2.98–6.43, *p* < 0.001).

The characteristics of the patients were analyzed according to the year of diagnosis in order to find an explanation for the SMR of the patients diagnosed after the year 2000, along with a worse prognosis observed within our cohort. The group of patients diagnosed after 2000 (*n* = 96) had more advanced stages (92% presented stages II + III + IV vs 80% in the group < 2000, *p* = 0.010), were older (17% ≥ 60 (*p* < 0.001) and were less responsive to treatment (no complete response 20% vs 5% in the <2000 group, *p* < 0.001) (Table 3).

**Table 3:** Patient characteristics according to the year of diagnosis (< 2000 vs ≥ 2000)

Characteristic	<2000	≥ 2000	*p*
	*N* (%)	*N* (%)	
Sex			0.626
Male	307 (62.02)	57 (59.38)	
Female	188 (37.98)	39 (40.63)	
Ann Arbor stage			0.081
I	96 (19.39)	7 (7.95)	
II	223 (45.05)	45 (51.14)	
III	113 (22.83)	23 (26.14)	
IV	63 (12.73)	13 (14.77)	
Age at diagnosis (years)			0.001
≤30	267 (54.05)	41 (42.71)	
31–59	199 (40.28)	39 (40.63)	
≥ 60	28 (5.67)	16 (16.67)	
Response to treatment			0.000
Complete	468 (94.93)	63 (79.75)	
Non complete	25 (5.07)	16 (20.25)	
Exitus			0.000
Yes	232 (47.15)	26 (27.08)	
No	260 (52.85)	70 (72.92)	
Cause of death			0.061
Primary tumor	48 (21.62)	8 (34.78)	
Secondary tumor	55 (24.67)	1 (4.35)	
Other causes	119 (53.60)	14 (60.87)	

## DISCUSSION

In Oncology, HL is usually presented as an example of curable disease due to its high number of DFS [6]. However, long-term survival studies have described that patients with HL do not have the same mortality compared to the general population, adjusted for age and sex [7]. In routine clinical practice, patient follow-up is focused on the early detection of complications such as the development of second malignancies, cardiac toxicities or other toxicities associated to treatment, which are commonly the cause of death [8].

In 1999, Provencio *et al*. [9] demonstrated an increased risk of mortality in patients with HL compared to the general Spanish population, 20 years after diagnosis.

In this study, we reported that even after almost 40 years from diagnosis of HL, patients triple the risk of death from all causes, compared to the general population. When studying the different causes of death, only 22.76% of them were due to the lymphoma itself. Therefore, the excess death observed in these patients is mainly due to other causes, primely second tumors, fatal cardiac events or other causes not related to the primary tumor). It is postulated that the risk of developing second malignancies is mainly due to complications derived from radiotherapeutic treatment used in HL, age, smoking history, and recently, it has been associated with a genetic predisposition [10, 11].

The risk of death was significantly higher in women than in men. However, in the internal analysis of our cohort, it was the men who had a higher risk of mortality by any cause, mainly due to presenting more advanced disease subtypes (lymphocyte depletion) at the time of diagnosis. Also of note, the great mortality of patients under 30 years of age (eleven-fold; SMR of 11.14), which could be justified by the fact that in the general population of the same age, the risk of death is very low, so a fatal event in our cohort would have a greater impact on the difference between observed and expected events.

In addition, and despite the advances achieved in the treatment of HL in the last decade, the risk of death was even greater in patients diagnosed after 2000. To understand the differences in mortality in relation to the time of diagnosis (before and after 2000), we compared both subgroups in our cohort. Patients diagnosed after the year 2000 had a worse prognosis, since there was a higher proportion of patients with advanced age (16.67% diagnosed after 2000 vs 5.67% diagnosed before 2000), and additionally had lower rates of complete response to initial treatment (20.25% vs 5.07%, respectively), both with statistical significance.

Therefore, we can conclude that in the Spanish population, HL currently presents a tendency towards more aggressive forms, with older age patients and a lower rate of complete response to first line treatments. There may be a bias regarding the transfer of complex patients to third-level hospitals such as ours and this may not represent the real situation of the global and current change in this lymphoma. In all, there may be an evident improvement in the average life expectancy in the Western population in the last decades and perhaps this could explain the differences in the risk of death. This is verified with a similar rate of OS at 20 years compared to that of other recently published series, which is around 65% [4].

The findings observed in this study may be of great relevance in the study of this disease, specially considering the inclusion of the large number of patients and the long follow-up. In addition, it was performed in a third-level hospital setting, a reference institution in HL, where most clinical records are computerized, which implies a reliable record of the patients’ medical history.

The interest of making future updates analyzing the hypotheses of this work is raised, since the development of new drugs, fundamentally based on anti-PD1 antibodies, such as nivolumab, has showed high response rates in adults with relapsed or refractory classical LH [12], which is expected to lead to a change in routine clinical practice.

Finally, this study is unique in the sense of analyzing the mortality of the patients comparing it to the Spanish population 40 years after diagnosis. Long-term follow-up of patients with HL may therefore focus on identifying late complications that constitute the leading cause of death in these patients.

## MATERIALS AND METHODS

### Patients

This study was performed at the Medical Oncology Department, at Puerta de Hierro University Hospital (Madrid, Spain). A total of 595 patients diagnosed with HL were included at our institution between January 1966 and February 2014. An anatomopathological confirmation of HL by a hematopathologist was considered mandatory for the inclusion of patients in the study.

The following variables were collected from the clinical records according to the standard hospital protocol: demographic data from each patient, clinical and pathological features, prognostic factors and treatments received type of response and survival, as well as the detection of specific causes of death.

All patients had to undergo an adequate extension study prior to initiation of treatment and during follow-up. In all cases, the stage was determined according to the Ann Arbor classification [13], later modified in 1988 at the Cotswolds meeting [14].

The following chemotherapy regimens were used: MOPP (clomiphene hydrochloride, vincristine sulfate, procarbazine hydrochloride and prednisone), ABVD (adriamycin, bleomycin, vinblastine and dacarbazine), a hybrid regimen of both, or other schemes in a small number of patients. Radiation therapy was performed following Kaplan’s field recommendations [15].

Information on the progression of disease or relapse, re-treatment, and death, as well as its causes, was verified through the analysis of medical health records, death certificates, or National Institute of Statistics (INE) records. The cause of death associated with HL included progression of the disease independently of other causes of death. Non-HL related deaths were included in those causes that occurred independent of lymphoma, secondary to the toxicities of chemotherapy treatments and development of secondary malignancies.

The study was reviewed and approved by the Ethics and Clinical Research Committee of Puerta de Hierro University Hospital.

### Statistical methodology

### Standardized mortality ratio analysis

Data from the Spanish general population were provided by the National Center for Epidemiology and registered by the INE from 1980 to 2013. Therefore, patients diagnosed from January 1980 were selected for this analysis. Patients with follow-up after December 31st 2013 were censored at this date. The standardized mortality ratio (SMR) is the ratio of the number of observed deaths (O) in our cohort versus expected (E) in the general Spanish population, adjusted for age, sex and time period. Both SMR and 95% confidence intervals (CI) were estimated on the hypothesis basis of the Poisson distribution for deaths observed in the follow-up period [16, 17]. Results were obtained for the entire cohort, segmented by gender, age groups at diagnosis (≤30, 31–59, and ≥60 years), and year of diagnosis (<2000 and ≥2000).

### Survival analysis of the entire cohort

All patients diagnosed with HL between 1966 and 2014 were included in this analysis. OS was defined as the time from diagnosis to the death of the patient; progression-free survival (PFS) was defined as the time from the end of treatment to relapse or death of the patient by any cause; and disease-free survival (DFS) as the time interval from first line treatment and a complete response, to relapse or death. If no events occured, observations were censored at the time of the last revision. The crude probability of the event was estimated using the Kaplan-Meier method, and the differences between the patient groups were assessed using the log-rank test.

A multivariate model of Cox proportional hazards was estimated, including sex, age at diagnosis (including three categories: ≤30 years, 31–59 years and ≥60 years), and diagnostic period (before and after 2000) to establish their association together in OS. The assumption of proportional hazards was assessed using Schoenfeld’s residues [18].

All statistical tests were two-tailed, and a p value <0.05 was considered statistically significant. All analyses were performed using Stata v14.2 (StataCorp, 2015. CollegeStation, TX: StataCorp LP).
